# Workplace resources, mentorship, and burnout in early career physician-scientists: a cross sectional study in Japan

**DOI:** 10.1186/s12909-020-02072-x

**Published:** 2020-06-03

**Authors:** Chithra R. Perumalswami, Shinichi Takenoshita, Ayumi Tanabe, Ranka Kanda, Haruko Hiraike, Hiroko Okinaga, Reshma Jagsi, Kyoko Nomura

**Affiliations:** 1 Center for Bioethics and Social Sciences in Medicine, University of Michigan Medical School, Ann Arbor, MI USA; 2 Department of Public Health, Teikyo University School of Medicine, Kaga 2-11-1, Itabashi-ku, Tokyo, 173-8605 Japan; 3 Department of Preventive Medicine and Public Health, Keio University School of Medicine, Shinanomachi 35, Shinjuku-ku, Tokyo, 160-8582 Japan; 4 Department of Obstetrics and Gynecology, Teikyo University School of Medicine, Kaga 2-11-1, Itabashi-ku, Tokyo, 173-8605 Japan; 5 Support Center for women physicians and researchers, Kaga 2-11-1, Itabashi-ku, Tokyo, 173-8605 Japan; 6 Department of Radiation Oncology and Center for Bioethics and Social Sciences in Medicine, University of Michigan, Ann Arbor, MI USA; 7 Department of Environmental Health Science and Public Health, Akita University Graduate School of Medicine, 1-1-1 Hondo, Akita, 010-8543 Japan

**Keywords:** Burnout, Workplace resources, Mentor, Physician-scientist, Grants

## Abstract

**Background:**

Physician-scientists are a vital segment of the healthcare workforce, but they may face significant challenges balancing and integrating clinical responsibilities, scientific research, and domestic responsibilities. This study investigates factors associated with burnout among highly successful early career physician-researchers in Japan.

**Method:**

Among 1790 physician awardees of Grant-in-Aid for Young Scientists by the Japanese Ministry in 2014–2015, 490 participated in this cross-sectional survey in 2016 (usable response rate 23.8%). The primary outcome was psychological burnout, measured by the Copenhagen Burnout Inventory (i.e., personal burnout, work-related burnout, and patient-related burnout). “Workplace resources” in our study refers to the presence of career education in the workplace, promotion of gender equity, well-being consultation services on “career and work,” “research,” “harassment,” and/or “mental health,” as well as the presence of a role model in the workplace who has perceived good work-life balance.

**Results:**

Among 408 physician-researchers (75% male, mean age 37 yrs), personal burnout scores were slightly higher in women than in men (mean score, 41.9 points vs. 36.7 points, difference, 5.2, 95% confidence interval, 0.5–9.9, *p* = 0.029), but work-related and patient-related burnout scores did not differ significantly between genders. Over half of women (64%) and men (58%) had a mentor (*p* = 0.374). In multivariable general linear regression models, personal burnout scores were higher for women (β = 4.98, *p* = 0.045), and lower among those who had a mentor (β = − 5.82, *p* = 0.010) and whose workplaces had well-being consultation services (β = − 0.79, *p* = 0.022). Work-related burnout scores were lower among those with larger amounts of grant funding (β = − 4.70, *p* = 0.013), a mentor (β = − 6.12, *p* = 0.002), well-being consultation services (β = − 0.78, *p* = 0.008) and a role model with a perceived good work-life balance (β = − 4.00, *p* = 0.038). Patient-related burnout scores were higher among physician-scientists aged older than 37 years (β = 6.25, *p* = 0.002) and those who had board certification (β = 9.01, *p* = 0.017), while these scores were lower among those had larger amounts of funding (β = − 5.01, *p* = 0.006) or a mentor (β = − 5.35, p = 0.006).

**Conclusions:**

Workplace resources and mentorship appear to be associated with lower levels of psychological burnout for both men and women early career physician-scientists.

## Introduction

Burnout is a pathological syndrome characterized by emotional exhaustion, reduced self-efficacy, and cynicism [1], which is primarily driven by workplace stressors [2]. Physician burnout in particular has reached epidemic proportions globally [3–6], with a prevalence of 43.9% in a recent national study of physicians in the United States of America [7]. Consequences are negative effects on professionals, patients, organizations, and society [7], including greater tendency towards future sickness, absence, and intention to quit [2].

Physician-scientists see patients and are also engaged in research. Research conducted by physicians is important because those with active clinical practices can directly translate insights from clinical experience to research questions and back into clinical practice, thereby rapidly accelerating the pace and impact of clinical translational research to improve human health [8, 9]. Although physician-scientists are a vital segment of the physician workforce, successful academic scientists face many barriers and require attainment of competitive grant funding, adequate research time, mentorship, success in publishing, as well as leadership skills [4, 10]. For example, in Japan with a small number of physicians (2.7 per 1000 population) compared to the average number of 3.3 among all Organisation for Economic Co-operation and Development (OECD) countries [11, 12], physician-scientists are extremely busy with daily clinical practice and have very limited protected time for research. Indeed, contributions to research published in the top general medicine journals from Japan have remained low over the last two decades [13]. Such a unique set of circumstances surrounding highly successful early career physician-scientists may incur tremendous psychological burden and dissatisfaction, resulting in greater intention to leave academia [14, 15].

In this study, we focused on an elite group of early career physician-scientists who are recipients of nationally sponsored, highly competitive research grants and are also actively involved in clinical practice. In a previous study of early- and mid-career physicians in Japan, we found that a research mentor or a Doctor of Medical Science degree is associated with an experience of successfully publishing original papers in peer-reviewed journals, which is both an indication of impact on scholarly discourse and an early indicator for later success [16]. Important challenges in balancing career and family demands for physicians have been demonstrated, particularly for women physicians [5, 17–21]. Much remains to be learned about the nature of challenges and impact of interventions on those pursuing careers in academic medicine [5], especially as there remains controversy about whether performing research is detrimental or protective to overall work-life satisfaction [22, 23]. Prior conceptual frameworks for burnout involve individual, organizational, and job stressors, each contributing to core attributes of burnout (emotional exhaustion, depersonalization, and diminished personal accomplishment) [1]. Hence, the primary purpose of this study was to investigate burnout among physician-scientists, as well as environmental factors (factors external to the self, such as those that are organizational or job-related) which might mitigate aspects of burnout, in order to further inform future organizational policies and practice [24–26]. The secondary purpose of this study is to further elucidate gender differences in burnout experienced by early career physician-scientists [5, 20, 21].

## Methods

### Grant-in-aid for young scientists in Japan

In this study, we focused on high-achieving physician-scientists who were recipients of the Grant-in-Aid for Young Scientists [27], which are very similar to prestigious K08 and K23 career development awards [28] from the United States National Institutes of Health. These highly competitive awards provide support for supervised research and study to early career clinically trained professionals who have the commitment and potential to develop into productive, independent scientific investigators [29].

### Participants

The authors identified 3143 recipients of the Grant-in-Aid for Young Scientists from 2014 to 2015 (S, A, or B awards) under the research area of medicine, excluding nursing science and dentistry, using the Kakenhi database (https://kaken.nii.ac.jp/ja/). Among these, we excluded 1353 awardees whose names were not registered in the medical license registration system (https://licenseif.mhlw.go.jp/search/jsp/top.jsp), a database of all medical license holders provided by Japanese Ministry. We then recruited the remaining 1790 awardees to participate by mail, and a total of 490 physician-scientist recipients agreed to participate in this study and returned questionnaires (usable response rate 23.8%). In total, we excluded 82 survey respondents as they had either withdrawn from the grant (51, with the majority of the reasons for withdrawal identified related to studying or working abroad), did not answer the self-reported question that verified medical licensure (56), or reported no involvement in clinical practice (26) at the time of response. As some of these individuals shared more than one of these exclusion criteria, the total number of eligible subjects left for further analyses was 408 from the original 490 respondents, out of 1713 from the eligible sample as those not seeing patients (77) were excluded from the sample. Therefore, our usable response rate (408/1713) was 23.8%.

This study was approved by the Teikyo University ethics committee (TU-COI 13–208).

## Measures

### Questionnaire

The questionnaire had 7 sections including 50 questions in total. Sections consisted of demographic information, physician experience and qualification, working conditions and research activities, organizational factors, mentorship, academic background during medical school, and burnout.

### Outcome/dependent variable

#### Copenhagen burnout inventory

This well-known, publicly available and previously validated inventory examines exhaustion and its attribution on three distinctive facets: personal burnout (general psychological and physical fatigue, 6 items), work-related burnout (7 items), and client-related burnout (“client” is heretofore replaced by “patient” in this study, 6 items) [2]. Each item was answered using a five-point Likert scale scoring: always for 100, often for 75, sometimes for 50, seldom for 25, and never/almost never for 0. The total score on the scale is the average of the scores on the items (i.e., the total score of each subscale is 100). If fewer than three questions of the personal and patient-related burnout subscales or if fewer than four questions of the work-related burnout subscale were answered, the respondent was classified as a non-responder. The three burnout subscale scores were calculated with one item reversed in order of response, so that higher overall scores indicated a greater degree of burnout.

#### Independent variable of interest

##### Workplace resources

The measure of “Workplace Resources” used in this study was based on a modified version of a checklist for a “female doctor-friendly workplace” that we previously developed using factor analysis [30]. The overall measure in that scale evaluates resources in a wide variety of contexts and seemed relevant for measurement in the context of evaluating initiatives to reduce burnout more generally. Because this original scale was developed primarily for promotion of gender equity among men and women, and was heavily weighted on gender, we conducted an exploratory factor analysis for the purpose of this study to investigate workplace resources for physician-scientists regardless of gender. Promax rotation identified two important factors: “Organizational climate” (Cronbach’s alpha coefficient .81) and “Well-being consultation services” (Cronbach’s alpha coefficient .74). “Organizational climate” concerns the meaning employees attach to the tangible policies, practices, and procedures they experience in their work situation [31]. It consists of three items—two relating to promotion of gender equity and one relating to career education in the workplace. The questions started with “Does your workplace have” followed by: “an effort to implement strategies to encourage males or the organization to combat gender inequalities?”, “seminars and workshops on gender equity for the entire organization?”, and “a curriculum on learning career development?”. “Well-being consultation services” consists of four items by asking if the workplace has someone or a division for consultation on “career and work”, “research”, “harassment”, and/or “mental health”. The response pattern was measured based on the physician-scientist level of agreement on a five-point Likert scale ranging from ‘strongly disagree’ to ‘strongly agree.’ Accordingly, “organizational climate” ranges 5–15 points and “well-being consultation services” ranges 5–20 points with higher scores indicating greater degrees of support in the workplace. Among four items which were dropped from the original scale by factor analyses, we retained one item related to the presence of a role model because the main purpose of our study is to investigate burnout and potential mitigating factors in a group of elite early career physician-scientists. We measured this item by asking “Does your workplace have a role model who can keep a good balance of career and personal life?” and the response was divided into a binary: ‘agree’ vs. either ‘disagree’ or ‘do not know.’ Hence, workplace resources in this study consists of three components including organizational climate, well-being consultation services, and a role model with a perceived good balance between work and life. The term workplace resources does not represent a comprehensive summary of the work environment or a comprehensive summary of all of the resources available in the workplace.

### Covariates

The items investigated in this present study included gender, age, years of experience as a physician, Doctor of Medical Science degree, board certification as a specialist, department (surgical, internal medicine, other clinical departments, and public health and basic medicine), amount of funding support, research type (i.e., basic medical science, clinical medicine, social medicine, others), overall weekly working hours and percent time devoted to various activities (patient care, research, education and career, teaching, administration, and others), marital status, household income, and the presence of a mentor (yes versus no or do not know), and workplace resources as defined previously.

### Data analyses

Baseline characteristics of the analytic sample are statistically tested according to gender using a t-test for continuous variables and a chi-square test or a Fisher’s exact test for categorical variables. Due to concerns about skewed data, analyses for continuous variables were repeated using nonparametric tests. These analyses yielded identical conclusions, so the parametric tests are reported throughout. In order to investigate factors associated with the three dimensions of burnout in the Copenhagen Burnout Inventory, general linear regression models were applied. Because of strong collinearity diagnosed in pre-analyses between age and years of experience as a physician, years of experience was eliminated from the linear regression analyses. We estimated a non-standardized regression coefficient with 95% confidence interval (95%CI), and the standard error for each variable in both univariate and multivariable models. The model selection in multivariable analyses used a forward stepwise method by using “glmselect procedure” in SAS software. We used a stepwise selection because among variables we considered, several variables had collinearity (for example, age and years of experience). For our secondary purpose of investigating gender differences in burnout, we investigated gender associations with burnout as well as any statistical interactions between gender and other covariates.

All analyses were conducted using SAS software Version 9.4 (Cary, NC), with statistical significance set at *p* < 0.05.

## Results

Table 1 shows baseline characteristics of the 408 physician-scientists in the analytic sample. Overall, 75% were male. Mean age was 37 years, and years of experience as a physician was approximately 12 years. Almost 90% of men were married as compared to 70% of women (*p* < 0.0001). For research type, a higher proportion of men were engaged in basic medicine or social medicine whereas a higher proportion of women were engaged in clinical medicine (*p* = 0.024). Men were more likely to report longer working hours than women (62.7 h vs. 50.0 h per week overall, *p* < 0.001). Distribution of working time also differed by gender; men reported spending more time on education and career (*p* = 0.022) and administrative work (p < 0.001). More than half of women (63%) and men (58%) answered that they had a mentor, and approximately 10% of both men and women answered that they do not know if they had a mentor. Among the three types of psychological burnout scores, the personal burnout score was significantly higher in women compared to men (41.9 vs. 36.7 points, difference, 5.2, 95% CI, 0.5–9.9,*p* = 0.029). Regarding workplace resources, more women than men reported that their workplace had well-being consultation services on “career and work”, “research”, “harassment”, and/or “mental health” (*p* = 0.004). Workplace resources and a role model with a perceived good work-life balance showed no statistically significant differences between women and men.

**Table 1:** Baseline characteristics of the analytic sample (408 Japanese physician-researchers awarded prestigious career development grants)

	Female (*n* = 101)	Male (*n* = 307)	*p* value^a^
Individual factor			
Age, years	36.6 ± 2.5	36.8 ± 2.7	0.585
Years of experience as a physician	12.2 ± 2.5	11.9 ± 2.7	0.338
Doctor of Medical Science, n (%)	77 (76.2)	220 (71.7)	0.370
Board certified specialist, n (%)	89 (88.17)	284 (92.8)	0.140
Specialty			0.258
Surgical departments	42 (41.6)	116 (37.8)	
Internal Medicine departments	32 (31.7)	124 (40.4)	
Other clinical departments	9 (8.9)	31(10.1)	
Public Health and Basic Medicine	18 (17.8)	36 (11.7)	
Grant-in-Aid for Young Scientists			
Type B	97 (99.0)	300 (98.7)	0.819
Amount of funding support ×100 US$	364 (140–741)	377 (156–500)	0.149
Research type, n (%)			0.024
Basic medicine	41 (42.7)	170 (55.9)	
Clinical medicine	51 (53.1)	114 (37.5)	
Social sciences in medicine/others	4 (4.2)	20 (6.6)	
Average weekly working hours	50.0 ± 16.7	62.7 ± 16.7	< 0.001
Attributes (%)			
Patient care	59.6 ± 24.5	54.4 ± 22.4	0.052
Research	22.0 ± 20.2	22.7 ± 19.2	0.733
Education and Career	4.5 ± 3.5	5.5 ± 4.6	0.022
Teaching	8.3 ± 10.2	7.5 ± 6.5	0.450
Administration	4.0 ± 5.2	8.5 ± 9.9	< 0.001
Others	4.0 ± 11.3	3.1 ± 4.7	0.489
Married, n (%)	74 (69.8)	293 (90.4)	< 0.001
Household income, US$(1$ = 100JPY), n (%)			0.128
≥ 200,000	19 (19.4)	40 (13.1)	
150,000-200,000	25 (25.5)	80 (26.2)	
100,000–150,000	36 (36.7)	146 (47.9)	
< 100,000	18 (18.4)	39 (12.8)	
Copenhagen Burnout Inventory (0–100 points)^b^			
Personal burnout (6 items)	41.9 ± 20.5	36.7 ± 20.8	0.029
Work-related burnout (7 items)	32.1 ± 15.3	31.9 ± 14.8	0.544
Patient-related burnout (6 items)	28.8 ± 20.5	27.8 ± 17.3	0.659
Mentor, n (%)^c^	64 (63.4)	178 (58.4)	0.374
Workplace Resources^d^			
Organizational climate (5–15 points)^e^	7.9 ± 3.0	8.4 ± 2.8	0.151
Well-Being consultation services^, f^ (5–20 points)	14.6 ± 3.3	13.4 ± 3.6	0.004
A role model with good work-life balance, n (%)^g^	36 (36.0)	127 (41.8)	0.307

Values are shown as number of physician-researchers (percentage), or mean ± S.D. for continuous variables whereas Grants-in-Aid for Young Scientists amount is shown as median (5–95% confidence intervals). Significance of the difference between male and female: *p* values were estimated based on a t-test, a chi-square test or a Fisher’s exact test^a^

^b^Each item was answered using a five-point Likert scale scoring: Always for 100, Often for 75, Sometimes for 50, Seldom for 25, and Never/almost never for 0. Total score on the scale is the average of the scores on the items (i.e., total score of each subscale is 100). The three burnout facet scores were calculated with one item reversed in order of response, so that higher overall scores indicate higher degree of burnout. ^c^The mentor item was counted comparing those who have a mentor vs. those who do not have a mentor or those who do not know if they have a mentor. ^d^Workplace Resources were asked starting with “Does your workplace have the following conditions....?” and response patterns were based on a five point Likert-scale from ‘strongly disagree’ to ‘strongly agree.’ ^e^Organizational climate includes the presence of actions to promote gender equity and presence of career education. ^f^Well-being consultation services measures if the workplace has an expert or a division for consultation on “career and work”, “research”, “harassment”, and “mental health”. ^g^ The item measures if the workplace has a role model who can keep a perceived good balance of career and personal life and divided into a binary variable (agree vs. either “do not know” or “disagree”

Table 2 shows factors associated with three types of burnout in univariable linear regression models. Factors that became statistically significant at 0.05 level were female gender for Personal burnout score (*p* = 0.029), and age for Patient-related burnout (p = 0.004), weekly working hour and Work-related burnout (*p* = 0.037), the presence of a mentor (all ps < 0.003), more supportive organizational climate (all ps < 0.031), well-being consultation services (all ps < 0.030), and a role model with a good work-life balance (all ps < 0.014).

**Table 2:** Factors associated with three types of burnout: univariable models

	Personal burnout^a^			Work-related burnout^a^			Patient-related burnout^a^		
	Estimate	SE	*P*	Estimate	SE	*P*	Estimate	SE	*P*
Individual factor									
Female	5.20 (0.54, 9.87)	2.37	0.029	1.27 (−2.77, 5.31)	2.05	0.537	1.00 (−3.09, 5.09)	2.08	0.630
Age, years									
37 - vs. < 37	1.82 (−2.29, 5.93)	2.09	0.385	1.89 (−1.65, 5.42)	1.80	0.294	5.30 (1.75, 8.84)	1.80	0.004
Doctor of Medical Science	1.94 (−2.61, 6.49)	2.31	0.400	0.43 (−3.49, 4.34)	1.99	0.831	1.98 (−1.98, 5.94)	2.02	0.330
Board certified specialist	−2.30 (−9.60, 5.01)	3.72	0.540	2.76 (−3.55, 9.06)	3.21	0.300	−0.74 (−7.12, 5.63)	3.24	0.820
Specialty vs. Public Health or Basic medicine			0.556			0.442			0.378
Surgery	4.51 (−1.94, 10.96)	3.28	0.170	3.92 (−1.63, 9.46)	2.82	0.166	3.13 (−2.48, 8.74)	2.86	0.274
Internal Medicine	4.23 (−2.23, 10.69)	3.29	0.199	3.90 (−1.65,9.46)	2.83	0.168	4.01 (−1.62, 9.63)	2.86	0.162
Other clinical departments	3.06 (−5.48, 11.59)	4.34	0.481	5.44 (−1.90,12.78)	3.73	0.146	6.25 (−1.18, 13.68)	3.78	0.100
Married	−2.96 (−8.84, 2.92)	2.99	0.320	−4.78 (−9.82, 0.27)	2.57	0.064	−1.90 (−7.03, 3.23)	2.61	0.470
Amount of Grant-in-Aid for Young Scientists ×100 US$(1$ = 100JPY)									
377 - vs. < 377	−1.77 (−5.85, 2.31)	2.07	0.395	−2.59 (−6.10, 0.91)	1.78	0.147	−3.20 (−6.74, 0.35)	1.80	0.077
Research type vs. social sciences in medicine/others			0.735			0.162			0.417
Basic medicine	3.42 (−5.36,12.22)	4.47	0.443	6.78 (−0.78, 14.33)	3.84	0.079	3.89 (−3.80, 11.59)	3.92	0.321
Clinical medicine	2.73 (−6.18, 11.64)	4.53	0.547	4.73 (−2.93, 12.40)	3.90	0.225	1.88 (−5.92, 9.69)	3.97	0.635
Average of weekly working hours	0.05 (−0.06, 0.17)	0.06	0.370	0.11 (0.01, 0.20)	0.05	0.037	0.01 (−0.09, 0.11)	0.05	0.860
Household income US$(1$ = 100JPY) vs. < 100,000			0.704			0.236			0.835
≥ 200,000	−3.23 (−10.77, 4.30)	3.83	0.399	−5.09 (−11.59, 1.40)	3.31	0.124	0.44 (−6.19, 7.06)	3.37	0.897
150,000-200,000	−2.21 (−8.88, 4.46)	3.39	0.515	−5.85 (11.61, −0.09)	2.93	0.046	−0.40 (−6.27, 5.47)	2.99	0.893
100,000–150,000	−3.58 (−9.74, 2.58)	3.13	0.253	−4.53 (−9.84, 0.79)	2.70	0.095	−1.67 (−7.09, 3.74)	2.75	0.544
Mentor	−6.89 (−10.97, −2.81)	2.08	0.001	−6.72 (−10.21, −3.22)	1.78	0.000	−5.40 (−8.95, − 1.85)	1.81	0.003
Workplace Resources^b^									
Organizational climate^c^	−0.82 (− 1.57, − 0.08)	0.38	0.031	− 0.89 (− 1.53, − 0.25)	0.32	0.007	−0.74 (− 1.40, − 0.08)	0.33	0.028
Well-Being consultation services^d^	− 1.10 (− 1.72, − 0.48)	0.31	0.001	−1.08 (− 1.61, − 0.55)	0.27	<.0001	−0.61 (− 1.15, − 0.06)	0.28	0.030
A role model^e^	−7.06 (− 11.16, − 2.30)	2.08	0.001	−5.82 (− 9.35, − 2.29)	1.80	0.001	− 4.54 (− 8.14, − 0.94)	1.83	0.014

^a^Each item was answered using a five-point Likert scale scoring: Always for 100, Often for 75, Sometimes for 50, Seldom for 25, and Never/almost never for 0. Total score on the scale is the average of the scores on the items (i.e., total score of each subscale is 100). ^b^Workplace Resources was asked starting with “Does your workplace have the following conditions....?” and response patterns were based on a five point Likert scale from ‘strongly disagree’ to ‘strongly agree.’ ^c^Organizational climate includes the presence of actions to promote gender equity and presence of career education. ^d^Well-being consultation services measures if the workplace has an expert or a division for consultation on “career and work”, “research”, “harassment”, and/or “mental health”. ^e^The item measures if the workplace has a role model who can keep a perceived good balance of career and personal life and divided into a binary variable (agree vs. either “do not know” or “disagree”).

Table 3 shows factors associated with three types of burnout in stepwise multivariable linear regression models. Personal burnout scores were higher for women (*p* = 0.045) and among physician-scientists who had a mentor (*p* = 0.010) and for those whose workplaces with well-being consultation services (*p* = 0.022). The work-related burnout score was lower among physician-scientists who had higher amount of grant (*p* = 0.013), a mentor (*p* = 0.008) and whose workplaces had well-being consultation services (p = 0.008) and a role model (*p* = 0.038). Patient-related burnout scores were higher in respondents whose age was older than 37 years (*p* = 0.002) and who had board certification (*p* = 0.017), and was lower among physician-scientists who had higher amount of grant (*p* = 0.006) and had a mentor (p = 0.006).

**Table 3:** Factors associated with three types of burnout: multivariable models.

	Personal burnout^a^ (R^2^ 8.0% *n* = 358)			Work-related burnout^a^ (R^2^ 11.7% *n* = 348)			Patient-related burnout^a^ (R^2^ 8.5% *N* = 349)		
	Estimate	SE	*P*	Estimate	SE	*P*	Estimate	SE	*P*
Individual factor									
Female	4.98 (0.12, 9.84)	2.47	0.045	–	–	–	–	–	–
Age, years									
37- vs. < 37	–	–	–	–	–	–	6.25 (2.38, 10.11)	1.97	0.002
Board certified specialist	–	–	–	–	–	–	9.01 (1.60, 16.42)	3.77	0.017
Married vs. single	–	–	–	−4.09 (−9.37, 1.19)	2.69	0.129	–	–	–
Amount of Grant-in-Aid for Young Scientists×100 US$(1$ = 100JPY)									
377 - vs. < 377	−3.34 (−7.57, 0.88)	2.15	0.120	−4.70 (−8.38, −1.02)	1.87	0.013	−5.01 (−9.16, − 1.55)	1.93	0.006
Research type vs. Social sciences in medicine /others	–	–	–			0.080	–	–	–
Basic medicine				7.61 (−0.20, 15.43)	3.97	0.056			
Clinical medicine				8.88 (1.12, 16.64)	3.94	0.025			
Mentor vs. none	−5.82 (−10.22, −1.42)	2.24	0.010	−6.12 (−9.90, −2.34)	1.92	0.002	−5.35 (− 9.16, − 1.55)	1.93	0.006
Workplace Resources^b^									
Organizational climate^c^	–	–	–	–	–	–	−0.54 (−1.23, 0.14)	0.35	0.120
Well-being consultation services^d^	−0.79 (−1.47, − 0.12)	0.34	0.022	− 0.78 (− 1.36, − 0.21)	0.29	0.008	–	–	–
A role model^e^	−3.82 (−8.20, 0.57)	2.23	0.088	−4.00 (−7.78, − 0.23)	2.08	0.038	−3.69 (− 7.67, 0.28)	2.02	0.068

^a^Each item was answered using a five-point Likert scale scoring: Always for 100, Often for 75, Sometimes for 50, Seldom for 25, and Never/almost never for 0. Total score on the scale is the average of the scores on the items (i.e., total score of each subscale is 100). ^b^Workplace Resources was asked starting with "Does your workplace have the following conditions....?" and response patterns were based on a five point Likert-scale from ‘strongly disagree’ to ‘strongly agree.’ ^c^Organizational climate includes the presence of actions to promote gender equity and presence of career education. ^d^Well-being consultation services measures if the workplace has an expert or a division for consultation on “career and work”, “research”, “harassment”, and/or “mental health”. ^e^The item measures if the workplace has a role model who can keep a perceived good balance of career and personal life and is divided into a binary variable (agree vs. either "do not know" or "disagree")

There were no statistically significant interactions found between gender and any of the other independent variables tested, including presence of a mentor and organizational support factors.

## Discussion

The impact of organizational leadership on physician well-being has recently been explored [32], with organizational factors and interventions found to result in clinically meaningful reductions in burnout [25, 26]. This study demonstrates that workplace resources and mentorship are associated with lower psychological burnout scores among highly successful early career physician-scientists. The presence of a mentor was associated with lower levels of all three burnout scores while the presence of well-being consultation services was associated with lower personal and work-related burnout scores. Having a role model with a perceived good work-life balance was associated with lower patient-related burnout. Female gender was associated with higher personal burnout scores, but there was no statistically significant interaction between gender and other variables studied, suggesting that mentorship and workplace resources are likely to be important for men and women alike.

Previous meta-analyses [33, 34] have suggested that burnout is a negative psychological response to prolonged stressors and correlates to low work performance and the intention to quit one’s job. In this regard, strategies in the workplace that alleviate psychological burden on physician-scientists are vital to retain physicians in both the clinical and academic workforce. Among the components of workplace resources we evaluated, well-being consultation services appeared to have a positive association with personal and work-related burnout while the presence of a role model in the workplace who had a perceived good work-life balance was interestingly associated with a lower score of patient-related burnout. A systematic review reported that perceptions of a good organizational climate were significantly associated with positive employee mental health outcomes such as lower levels of burnout, depression, and anxiety [35]. This review specifically highlighted the importance of social support from a supervisor or coworkers in the workplace. The role of social support in the workplace has long been studied by using the job demand-control-support model [36]. By contrast, the current study underscores the importance of a generally supportive atmosphere in the workplace. The presence of a mentor in the workplace appeared to be a robust, independent factor associated with less psychological burnout. To the extent that effort spent in mentorship is currently not recognized or rewarded at many academic institutions, this finding suggests the importance of finding ways to reward this critical activity. It should be noted that approximately 40% of our study subjects reported either not having mentor or not knowing if they had a mentor. Another study in Japan that investigated 683 physician-scientists demonstrated that most (91%) reported that they had a departmental mentor [37]. One explanation of the difference is contextual: as in many other academic institutions around the world, everyone who belongs to academic institutions in Japan has departmental leaders who may serve in a mentorship capacity, but those who indicated having a mentor in the current study are likely to be those with more formal, defined research mentoring relationships due to their attainment of competitive grant awards that support research and career development. Therefore, more emphasis on career development and formal mentor training might be considered, as the association of mentorship with burnout observed in this study might be because mentorship may improve self-efficacy to combat psychological stress and improve retention in academic careers.

Because gender equity issues are prevalent in academia and in medicine, the finding of gender differences in personal burnout scores merit further attention. In Western countries as well, women tend to bear the greater burden of domestic responsibilities [38], be significantly underrepresented in research [39], receive less research funding [35], and appear less frequently than men as authors on research publications [40]. Interestingly, women physicians in the US often have lower salaries than their male counterparts [41], but also provide better hospital care for the elderly [42]. A study of 1066 recipients of NIH K-awards demonstrated that women were also more likely to experience gender bias and sexual harassment in their careers [43]. In Japan, the situation appears even worse (the gender gap index of Japan ranks at 111th/144 countries in 2016), and the low proportion of women included in this elite sample is evidence of that concern, as is the greater level of personal burnout we observed among women physician-scientists. Just as in many Western studies of similar populations, most participants were married, but women were more likely to be single than men, suggesting that in order to be productive in the workplace (and in particular in academia), women might be more likely to require sacrifice in their personal lives with respect to foregoing marriage or delaying child-bearing [17, 44]. Nevertheless, it is important to emphasize that although burnout scores differed by gender, the positive association between workplace resources and mentorship was observed among men and women alike in this study.

Several limitations in the current work should be addressed. First, because our survey was cross-sectional, caution should be employed when making causal inferences. Second, non-response bias may exist if those who chose to respond to our survey differed meaningfully from those who did not. Because this study was conducted as part of a broader research project evaluating gender equity and the support of women, some respondents might have been particularly interested in that topic. Our low usable response rate at 23.8% makes this sort of response bias a distinct possibility. Third, 75% of our study participants were men. However, according to a Survey of Physicians, Dentists and Pharmacists conducted by Japanese ministry in 2014, the gender ratio of males to females in physicians is 4:1. Furthermore, recent OECD health data reported that the proportion of women physicians in Japan is the lowest among OECD countries [12]. Hence, although we do not have similar exact statistics for physician-scientists in Japan, we believe the percentage of male respondents (75%) in this study closely reflects the actual gender ratio in this population in which men far outnumber women. Fourth, psychological status may vary according to events associated not only with work but also in one’s personal life, and the current study did not include measures of experiences such as the presence of children or caregiving for elderly parents. Fifth, it is possible that some other underlying confounding factor leads to the observed associations. For example, although the clinical department was not significant in our study, the department discipline and resources might be potentially associated. Indeed, R-squared statistics ranged from 7.1 to 13.4%, indicating that only approximately 10% of the dependent variable was explained by the independent variables in the models. Careful interpretation of our results is therefore required, and a detailed understanding of the underlying sociodemographic characteristics and workplace environment (which was not thoroughly measured in our study) represents a step forward in this regard.

## Conclusion

In their efforts to mitigate burnout and help early career men and women physician-scientists achieve their full potential, academic medical centers might consider incorporating workplace resources and formal mentoring programs in their institutional policies. In the US, the Accreditation Council for Graduate Medical Education has recently required programs to promote wellness and monitor burnout among postgraduate medical trainees [45]. Similar types of policies might be useful for early career physician-scientists in Japan. Gender differences in personal burnout merit further attention as developing and maintaining a resilient physician scientist workforce is vital to providing both high quality health care and translating research innovations to the bedside.

## Data Availability

The datasets generated and/or analyzed during the current study are not publicly available due to protecting the privacy of participants but are available in anonymized format from the corresponding author upon reasonable request.
